# Contributions of Dickkopf‐1 to Obesity‐Induced Bone Loss and Marrow Adiposity

**DOI:** 10.1002/jbm4.10364

**Published:** 2020-04-28

**Authors:** Juliane Colditz, Ann‐Kristin Picke, Lorenz C. Hofbauer, Martina Rauner

**Affiliations:** ^1^ Department of Medicine III, Center for Healthy Aging Technische Universität Dresden Dresden Germany

**Keywords:** ADIPOCYTE, BONE MARROW ADIPOSITY, BONE MASS, DICKKOPF‐1, HIGH‐FAT DIET, OSTEOBLAST

## Abstract

Low bone strength in overweight individuals is a significant medical problem. One important determinant of mesenchymal stem cell fate into osteoblasts or adipocytes is the Wnt signaling pathway. We recently showed that Dickkopf‐1 (DKK1), a potent Wnt inhibitor, is upregulated in obese mice. In this study, we investigated the role of DKK1 in the pathogenesis of obesity‐induced bone loss using global and tissue‐specific KO mice. Obesity was induced in 8‐week‐old male mice with an inducible global (Rosa26‐CreERT2) or osteoprogenitor‐ (Osx–Cre‐) specific deletion of *Dkk1* with a high‐fat diet (HFD) containing 60% fat. After 12 weeks, body weight, bone volume, bone fat mass, and bone turnover were assessed. *Dkk1* ^*fl/fl*^ *;Rosa26‐CreERT2* mice experienced a similar increase in body weight and white fat pads as control mice. A HFD significantly reduced trabecular bone mass and the bone formation rate in Cre‐ mice and *Dkk1* ^*fl/fl*^ *;Rosa26‐CreERT2* mice. Interestingly, *Dkk1* ^*fl/fl*^ *;Rosa26‐CreERT2* mice were protected from HFD‐induced cortical bone loss. Furthermore, a HFD was associated with increased bone marrow fat in the femur, which was less pronounced in *Dkk1* ^*fl/fl*^ *;Rosa26‐CreERT2* mice. Mice with an osteoprogenitor‐specific *Dkk1* deletion showed similar results as the global knockout, showing a protection against HFD‐induced cortical bone loss and an accumulation of bone marrow fat, but a similar decrease in trabecular bone volume. In summary, DKK1 appears to contribute distinctly to cortical, but not trabecular bone loss in obesity. © 2020 The Authors. *JBMR Plus* published by Wiley Periodicals, Inc. on behalf of American Society for Bone and Mineral Research.

## Introduction

Obesity is a worldwide health problem with more than 1.9 billion adults overweight in 2014 and over 650 million people obese.^1^ These numbers are predicted to increase to 2.16 billion overweight and 1.12 billion obese adults by 2030.^2^ Besides causing a high economic burden, the consequences of overweight and obesity on health are manifold. Overweight is a risk factor for type 2 diabetes mellitus as well as heart and vascular diseases leading to increased mortality.^3^ In addition, overweight affects bone metabolism and reduces bone strength.^4^

Previously, high‐body weight was considered protective against the development of osteoporosis, which is characterized by decreased bone quality and increased fracture risk,^4^, ^5^ as BMI is positively correlated with BMD. However, recent studies show that an elevated abdominal fat tissue and BMI are relevant risk factors for osteoporosis in both sexes.^4^, ^6^, ^7^, ^8^ Moreover, although fracture risk is frequently reported to be lower at the proximal femur and vertebra in obese adults, it is increased at the proximal humerus, upper leg, and ankles.^9^ Therefore, obesity is not per se protective against fractures.

Mechanistically, the positive association of BMI and BMD may stem from increased mechanical loading and enhanced aromatase activity in fat tissue^10^, ^11^ as well as the bone‐anabolic action of fat‐derived hormones.^12^, ^13^ However, visceral fat is also associated with inflammation as reflected by the higher secretion of proinflammatory cytokines such as TNFα and IL‐6, which negatively affect bone metabolism.^14^, ^15^, ^16^ Finally, obesity leads to an increase in bone marrow fat, a condition that usually occurs in aged individuals.^17^, ^18^, ^19^, ^20^, ^21^, ^22^ The increase in bone marrow adiposity may indicate a shift in the fate decision of mesenchymal stem cells (MSCs) from osteoblasts to adipocytes,^23^ thereby increasing fat accumulation and reducing osteoblastic activity. The Wnt signaling pathway plays an important role in this process as Wnt activation supports osteoblastogenesis and at the same time, inhibits adipogenesis.^24^ Wnt6, Wnt10, and Wnt10b especially contribute to MSC fate decision via β‐catenin‐dependent mechanisms.^25^ Recently, Dickkopf‐1 (DKK1) and sclerostin (SOST), two important Wnt inhibitors, have emerged as promising new targets for anabolic therapies.^26^ *Dkk1* expression was shown to be transiently upregulated during adipogenesis in humans and correlated with an inhibition of the canonical Wnt signaling.^27^ Furthermore, *Dkk1* overexpression promotes adipogenesis,^27^ whereas siRNA‐mediated knockdown of *Dkk1* inhibits adipogenesis.^28^ Both global deletion of SOST and SOST antibody treatment resulted in an increased trabecular bone volume and a decrease in the number of bone marrow adipocytes, as well as a decrease in adipocyte size.^29^

Recently, we and others demonstrated that serum and skeletal levels of DKK1, but not SOST, are elevated in obese mice^20^, ^30^ and in patients with type 2 diabetes mellitus.^31^, ^32^, ^33^ As DKK1 is a potent suppressor of bone formation and bone mass,^34^, ^35^, ^36^ we hypothesized that elevated DKK1 levels may drive obesity‐induced bone loss in mice. To test this hypothesis, we fed *Dkk1* ^*fl/fl*^ *;Rosa26‐ERT2‐Cre* and *Dkk1* ^*fl/fl*^ *;Osx‐Cre* mice with a high‐fat diet (HFD) and analyzed bone mass and bone metabolism, as well as bone marrow adiposity. We found that DKK1 plays a site‐specific role in obesity‐induced bone loss in mice, contributing to cortical, but not trabecular bone loss.

## Methods

### Mice

For global *Dkk1* deletion, tamoxifen‐inducible global *Dkk1* KO mice (*Dkk1* ^*fl/fl*^ *;Rosa26‐CreERT2*) were generated.^34^ At the age of 5 weeks, male *Dkk1* ^*fl/fl*^ *;Rosa26‐CreERT2*‐positive and negative control mice were injected i.p. with 100 μL tamoxifen (10 g/L; Sigma, Merck KGaA, Darmstadt, Germany) for 5 consecutive days to induce global deletion of *Dkk1*. For cell‐specific deletion of *Dkk1* in osteoprogenitor cells, doxycycline‐repressible *Dkk1* ^*fl/fl*^ *;Osx‐Cre* mice were generated as previously reported.^34^ *Dkk1* ^*fl/fl*^ *;Osx‐Cre* breeding pairs received doxycycline in their drinking water (10 mg/mL in a 3% sucrose solution) *ad libitum* to repress Cre activity during embryogenesis. *Dkk1* ^*fl/fl*^ *;Osx‐Cre* offspring received doxycycline drinking water until the age of 5 weeks. Respective Cre‐negative littermates were used as controls. By suppressing Cre activity during embryogenesis, no effects on bone were observed in *Dkk1* ^*+/+*^ *;Osx‐Cre +* mice.^36^ Breeding of the mouse lines was approved by the institutional animal care committee of the Technische Universität (TU) Dresden and the Landesdirektion Sachsen.

### In vivo experiments

All animal procedures were approved by the institutional animal care committee of the TU Dresden and the Landesdirektion Sachsen. All mice were fed a standard diet with water *ad libitum* and were kept in groups of four animals per cage for the whole experiment. Mice were exposed to a 12‐hour light/dark cycle in an air‐conditioned room at 23°C (no specific pathogen‐free room), and housed in cardboard houses with bedding material. Mice were randomly assigned to treatment groups; subsequent analyses were performed in a blinded fashion. Male mice are commonly used for HFD interventions^37^, ^38^, ^39^ and were therefore chosen for all experiments. To mimic an excess uptake of fat, mice were fed a HFD (60% fat, 20% carbohydrate, and 20% protein; Research diets #12492, Research Diets, Inc., New Brunswick, NJ, USA) at the age of 8 weeks for 12 weeks. Control mice continued to receive the normal diet (ND: 9% fat, 58% carbohydrates, and 33% protein; Sniff #V1534‐300, Research Diets, Inc., New Brunswick, NJ, USA). Animal cohort sizes were as follows: *Dkk1* ^*fl/fl*^ *;Rosa26‐CreERT2*: 104 Cre‐negative ND: 12, Cre‐negative HFD: 10, Cre‐positive ND: 8, Cre‐positive HFD: 10 and *Dkk1* ^*fl/fl*^ *;Osx‐Cre*: Cre‐negative ND: 14, Cre‐negative HFD: 12, Cre‐positive ND: 12, Cre‐positive HFD: 10. Weight and blood glucose were measured every 4 weeks.

Additional methods (glucose tolerance test; assessment of bone mass, microarchitecture, and fat content; histology; histomorphometry; RNA isolation; qRT‐PCR; and statistics) are reported in the online Supplemental Material.

## Results

### HFD increases adiposity regardless of DKK1 expression

**Figure 1:**
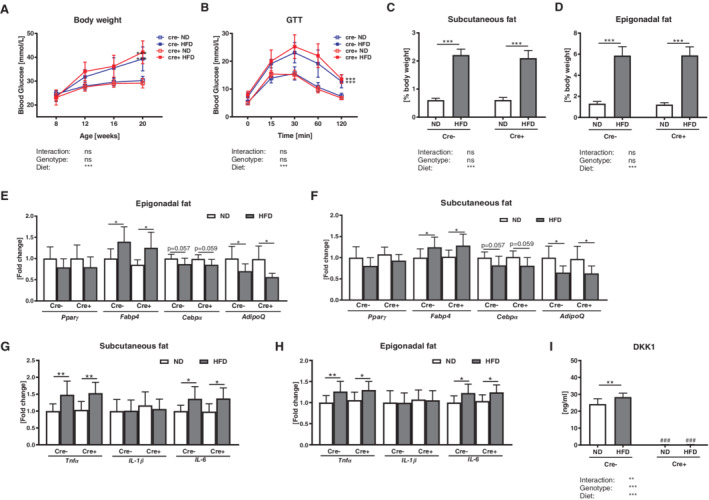
Global *Dkk1* cKO mice show similar signs of obesity, despite reduced DKK1 serum levels. Male *Dkk1* ^*fl/fl*^ *;Rosa26‐CreERT2* (Cre‐positive) and their Cre‐negative were fed a standard (ND) or high fat diet (HFD) for 12 weeks. Afterwards (*A*) body weight was assessed and (*B*) a glucose tolerance test (GTT) was carried out. Percentage of body (*C*) subcutaneous and (*D*) epigonadal fat mass was determined. Peroxisome proliferator‐activated receptor gamma (*Pparγ*), fatty acid binding protein (*Fabp4*), CCAAT/enhancer‐binding protein alpha (*Cebpα*), and adiponectin (*AdipoQ*) mRNA expression in (*E*) subcutaneous and (*F*) epigonadal fat mass was analyzed using real‐time PCR analysis. Gene expression of inflammation markers *Tnfα*, *Il‐1β*, and *Il‐6* in (*G*) subcutaneous and (*H*) epigonadal fat mass was analyzed. (*I*) Serum Dickkopf‐1 (DKK1) levels were assessed using commercially available ELISAs. Gene expression levels were normalized to *Rpl26*. Data represent the mean ± SD (*n* = 8 to 12/group). Statistical analysis was performed by two‐way ANOVA for the effect of genotype and HFD and the interaction. For weight and GTT area under the curve was determined. *p < 0.05, **p < 0.01, ***p < 0.001 for ND versus HFD. #*p* < 0.05, ##*p* < 0.01, ###*p* < 0.001 versus respective Cre‐negative control.

To assess the contribution of DKK1 to the pathogenesis of obesity‐induced bone loss, we subjected *Dkk1* ^*fl/fl*^ *;Rosa26‐CreERT2* (global *Dkk1* cKO) mice to a HFD for 12 weeks. *Dkk1* ^*fl/fl*^ *;Rosa26‐CreERT2* and Cre‐negative control mice gained a similar amount of weight when fed a HFD (40% to 45%), whereas mice fed a ND only gained 16% to 19% body weight after 12 weeks (Fig. 1 *A*). Furthermore, the HFD decreased glucose tolerance in both genotypes (Fig. 1 *B*).

The HFD‐induced gain in body weight in both genotypes was accompanied by a significant increase in their percentage of body subcutaneous and epigonadal fat mass compared with mice fed a ND (Fig. 1 *C*,*D*). Gene expression analysis revealed an increased fatty acid binding protein (*Fabp4*) and reduced adiponectin (*AdipoQ*) expression after a HFD in subcutaneous and epigonadal adipose tissue of *Dkk1* ^*fl/fl*^ *;Rosa26‐CreERT2* and Cre‐negative control mice, whereas *Pparγ* and *Cebpα* expression was not altered (Fig. 1 *E*,*F*). As obesity also causes low‐grade chronic inflammation,^40^, ^41^ we investigated the expression of the inflammation markers *Tnfα*, *Il‐1β*, and *Il‐6* in subcutaneous and epigonadal fat. *Tnfα* and *Il‐6* expression was similarly increased in global as well as control mice fed a HFD, whereas *Il‐1β* was not altered (Fig. 1 *G*,*H*).

As DKK1 was recently shown to be upregulated in bone and serum after a HFD in mice, we analyzed DKK1 serum levels.^20^ In accordance with this study, DKK1 serum levels were significantly increased in control littermates after a HFD (+15%), whereas *Dkk1* ^*fl/fl*^ *;Rosa26‐CreERT2* mice showed depleted DKK1 serum levels (Fig. 1 *I*). SOST serum levels were not altered after a HFD (Cre‐negative: ND: 166 ± 10, HFD: 172 ± 14, Cre‐positive: ND: 192 ± 16, HFD: 198 ± 14), albeit DKK1‐deficient mice showed an overall increased SOST serum level as compared with Cre‐negative.

### DKK1 may contribute to the HFD‐induced increase in bone marrow adiposity

**Figure 2:**
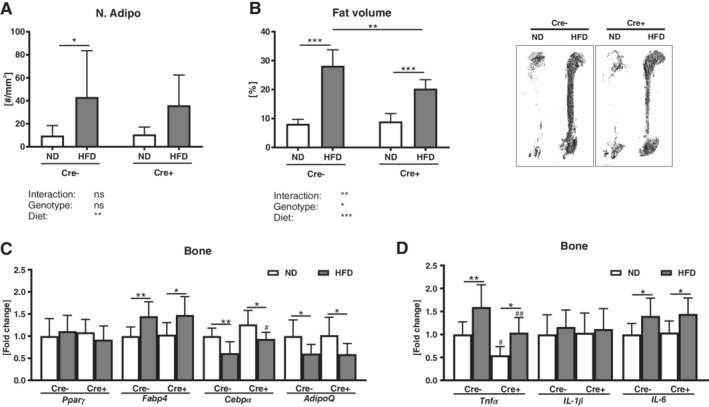
Global Dickkopf‐1 (*Dkk1*) deletion exhibit diminished expansion of bone marrow adipose tissue after HFD. *Dkk1* ^*fl/fl*^ *;Rosa26‐CreERT2* and their Cre‐negative controls were fed a normal (ND) or high fat diet (HFD) for 12 weeks. The amount of fat in the tibiae was assessed by counting (*A*) the number of adipocytes on tartrate‐resistant acid phosphatase‐ (TRAP‐) stained tissue sections and by (*B*) osmium staining of the whole femur. (*C*) Peroxisome proliferator‐activated receptor gamma (*Pparγ*), fatty acid binding protein (*Fabp4*), CCAAT/enhancer‐binding protein alpha (*Cebpα*), and adiponectin (*AdipoQ*) mRNA expression in ulnas was analyzed using real‐time PCR analysis. (*D*) Gene expression of inflammation markers *Tnfα*, *Il‐1β*, and *Il‐6* in ulnas was analyzed. Gene expression levels were normalized to *β‐Actin*. Data represent the mean ± SD (*n* = 8 to 12/group). Statistical analysis was performed by two‐way ANOVA for the effect of genotype and HFD and the interaction. *p < 0.05, **p < 0.01, ***p < 0.001 for ND versus HFD. #*p* < 0.05, ##*p* < 0.01, ###*p* < 0.001 versus respective Cre‐negative control.

Obesity is associated with an accumulation of fat in the bone marrow. Thus, we counted adipocyte ghosts on tissue sections of the tibia and performed osmium staining on the femur. A HFD increased the adipocyte number (+70–77%) and adipocyte area (+59–77%) in both genotypes (Fig. 2 *A* and Table 1). However, analyzing the entire bone marrow fat content using μCT, bone marrow fat tissue in global cKO mice was increased to a smaller extent than in their WT littermate controls (Fig. 2 *B*). Although *Fabp4* and *AdipoQ* showed a similar regulation after HFD in both genotypes, the reduction of *Cebpα* expression after HFD was less pronounced in global cKO mice (Fig. 2 *C*). *Pparγ* expression was not affected by a HFD. Similar to fat tissue, a HFD increased the expression of the inflammation markers *Tnfα* and *Il‐6* in both groups (Fig. 2 *D*). Furthermore, global cKO mice fed a ND exhibited a lower *Tnfα* expression, when compared with littermate controls.

**Table 1:** Bone Microstructure and Histological Parameters of Femurs and Tibias of 20‐Week‐Old *Dkk1* ^*fl/fl*^ *;Rosa26‐CreERT2* Mice

	Cre‐negative			Cre‐positive					
	ND (*n* = 12)	HFD (*n* = 10)	% change	ND (*n* = 8)	HFD (*n* = 10)	% change	Interaction	Genotype	Diet
*Dkk1* ^*fl/fl*^ *;Rosa26‐CreERT2*									
μCT—femur									
BV/TV (%)	14.2 ± 1.42	11.9 ± 1.53	−16%	20.0 ± 2.29***	17.9 ± 2.24***	−11%	0.930	<0.001	<0.01
Tb.N (1/mm)	4.44 ± 0.46	4.05 ± 0.21	−9%	4.95 ± 0.35	4.61 ± 0.48	−7%	0.843	<0.001	<0.01
Tb.Th (μm)	45.8 ± 3.55	42.8 ± 1.27	−7%	51.2 ± 2.15	48.6 ± 2.52*	−5%	0.363	0.212	<0.05
Tb.Sp (mm)	0.21 ± 0.02	0.24 ± 0.02	+15%	0.19 ± 0.01	0.21 ± 0.02**	+10%	0.230	<0.001	<0.001
Histomorphometry—tibia									
Adipo. Ar (mm^2^)	2.85 ± 1.71	12.5 ± 7.79	+77%	3.73 ± 3.10	9.03 ± 9.90	+59%	0.647	0.729	<0.01
BFR/BS (μm^3^/μm^2^/d)	0.35 ± 0.06	0.27 ± 0.07	−23%	0.47 ± 0.07***	0.40 ± 0.05***	−15%	0.441	<0.001	<0.001
MS/BS (%)	24.7 ± 2.15	19.6 ± 4.46	−21%	25.3 ± 2.18	22.4 ± 2.42	−15%	0.298	0.111	<0.001
MAR (μm/d)	1.42 ± 0.23	1.38 ± 0.23	−3%	1.86 ± 0.29**	1.79 ± 0.16**	−4%	0.876	<0.001	0.481
N.Oc/B.Pm (#/mm)	6.35 ± 2.27	6.44 ± 3.07	+2%	8.08 ± 3.75	7.46 ± 3.05	−8%	0.839	0.408	0.693
N.Ob/B.Pm (#/mm)	5.02 ± 1.63	3.72 ± 1.10	−26%	6.00 ± 1.61	4.20 ± 1.31	−30%	0.613	0.137	<0.01

Data represent the mean ± SD (*n* = 10 to 14/group). Statistical analysis was performed using two‐way ANOVA. *p* Values from ND versus HFD. *p < 0.05, **p < 0.01, ***p < 0.001 versus respective Cre‐negative control. BV/TV = bone volume/total volume; Tb.N = trabecular number; Tb.Th = trabecular thickness; Tb.Sp = trabecular separation; Adipo. Ar = adipocyte area; BFR/BS = bone formation rate/bone surface; MS/BS = mineralizing surface/bone surface; MAR = mineral apposition rate; N.Oc/B.Pm = number of osteoclasts/bone perimeter; N.Ob/B.Pm = number of osteoblasts/bone perimeter.

### Global DKK1 deletion does not protect against obesity‐induced cancellous bone loss, but protects cortical bone loss

**Figure 3:**
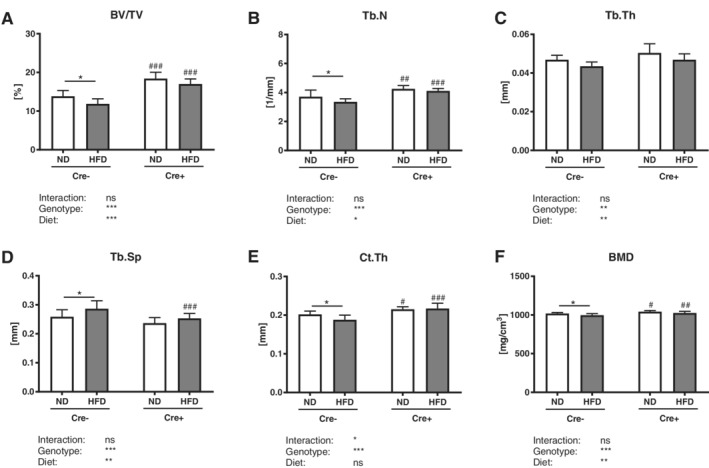
Global *Dkk1* deletion does not protect against obesity‐induced cancellous bone loss, while it modifies cortical thinning after HFD. The fourth vertebral body of 20‐week‐old male *Dkk1* ^*fl/fl*^ *;Rosa26‐CreERT2* (Cre‐positive and Cre‐negative) mice after 12 weeks of normal (ND) or high fat diet (HFD) was analyzed by μCT. (*A*) Trabecular bone volume per total volume (BV/TV) and (*B*) trabecular number (Tb.N), (*C*) trabecular thickness (Tb.Th) and (*D*) trabecular separation (Tb.Sp) of the fourth vertebral body. Cortical compartment of the femur was analyzed by μCT to examine (*E*) cortical thickness (Ct.Th) and (*F*) BMD of the femur. Data represent the mean ± SD (*n* = 8 to 12/group). Statistical analysis was performed by two‐way ANOVA for the effect of genotype and HFD and the interaction. *p < 0.05, **p < 0.01, ***p < 0.001 for ND versus HFD. #*p* < 0.05, ##*p* < 0.01, ###*p* < 0.001 versus respective Cre‐negative control.

*Dkk1*‐proficient mice showed a significant reduction of vertebral trabecular bone volume (−14%) and trabecular number (−10%) after a HFD, whereas trabecular thickness was not affected (Fig. 3 *A–C*). Furthermore, Cre‐negative mice showed a significant increase in trabecular separation (+10%; Fig. 3 *D*). Similarly, global *Dkk1* cKO mice lost vertebral bone structure, even though to a smaller extent (Fig. 3 *A*–*D*). Similar results were found in the femur (Table 1). However, in cortical bone, only Cre‐negative HFD mice exhibited a significantly reduced cortical thickness (−7%) and BMD (−3%), whereas global cKO mice were protected from bone loss (Fig. 3 *E*,*F*).

### Global DKK1 deletion tends to reduce the HFD‐mediated reduction of bone formation

**Figure 4:**
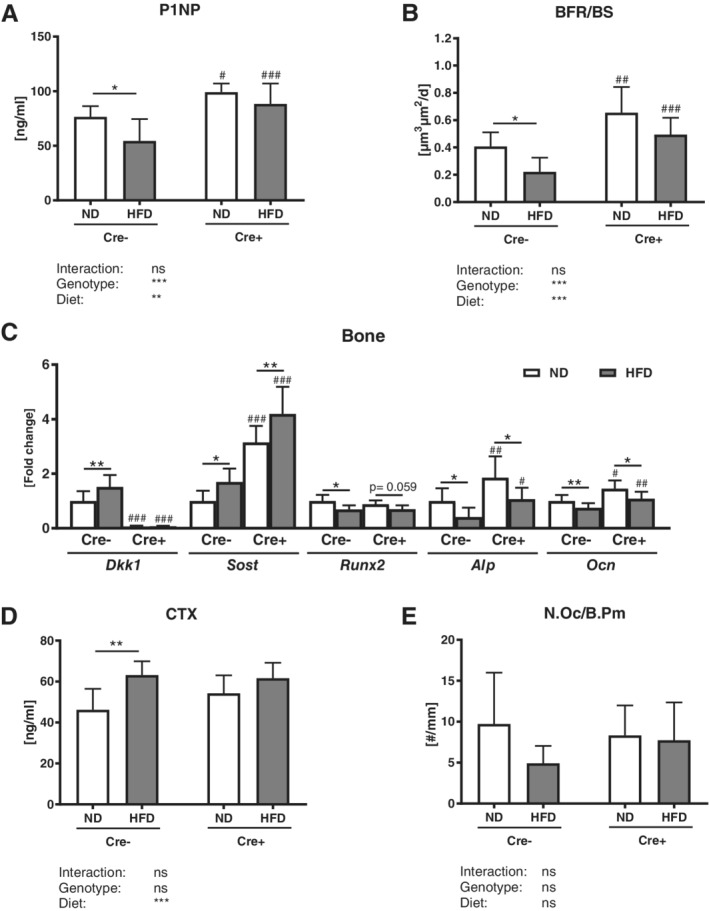
Global Dickkopf‐1 (*Dkk1*) cKO mice show no alterations in bone formation and bone resorption after a HFD. Histomorphometric and serum osteoblast and osteoclast parameters of 20‐week‐old male *Dkk1* ^*fl/fl*^ *;Rosa26‐CreERT2* (Cre‐positive and Cre‐negative) mice after 12 weeks of normal (ND) or high‐fat diet (HFD) were analyzed. (*A*) Quantification of serum procollagen type 1 amino‐terminal propeptide (P1NP) was performed by ELISA. Histomorphometric analysis of calcein double staining of tibias was performed to determine (*B*) the bone formation rate/bone surface (BFR/BS). (*C*) Dickkopf‐1 (*Dkk1*), sclerostin (*SOST*), Runt‐related transcription factor 2 (*RUNX2*), alkaline phosphatase (*Alp*), and osteocalcin (*Ocn*) mRNA expression in ulna bone tissue was analyzed using real‐time PCR analysis. Gene expression levels were normalized to *β‐actin*. (*D*) Serum CTx was measured using ELISA. (E) Tartrate‐resistant acid phosphatase (TRAP) staining was used to determine the number of osteoclasts/bone parameter (N.Oc/b.pm) in tibias. Data represent the mean ± SD (*n* = 8 to 12/group). Statistical analysis was performed by two‐way ANOVA for the effect of genotype and HFD and the interaction. *p < 0.05, **p < 0.01, ***p < 0.001 for ND versus HFD. #*p* < 0.05, ##*p* < 0.01, ###*p* < 0.001 versus respective Cre‐negative control.

The HFD in control mice led to a reduction of P1NP serum levels (−28%), a reduced mineral surface per bone surface (−21%), a reduced number of osteoblasts (−32%), and a decreased bone formation rate (−31%) in the appendicular skeleton, whereas the mineral apposition rate (MAR) was not altered (Fig. 4 *A*,*B* and Supplemental Table S2). All these parameters were reduced to a lower extent in global *Dkk1* cKO mice, yet the interaction effect did not show a significant difference (Fig. 4 *A*,*B* and Supplemental Table S2). Similar results were found in the tibias of the mice (Table 1). Local *Dkk1* expression was significantly increased in Cre‐negative control mice fed a HFD when compared with a ND, and was not detectable in mice with a global *Dkk1* deletion (Fig. 4 *C*). SOST expression was increased under HFD conditions and was further increased in global cKO bones (Fig. 4 *C*). Runt‐related transcription factor 2 (*RUNX2*), alkaline phosphatase (*Alp*), and osteocalcin (*Ocn*) expression was significantly reduced after a HFD in both genotypes.

Furthermore, CTx serum levels were significantly increased (+27%) after HFD in control mice, but not in *Dkk1* cKO mice (Fig. 4 *D*). The number of osteoclasts was not altered by a HFD (Fig. 4 *E*).

### Osteoprogenitor‐specific DKK1 deletion does not protect against HFD‐induced adiposity

**Figure 5:**
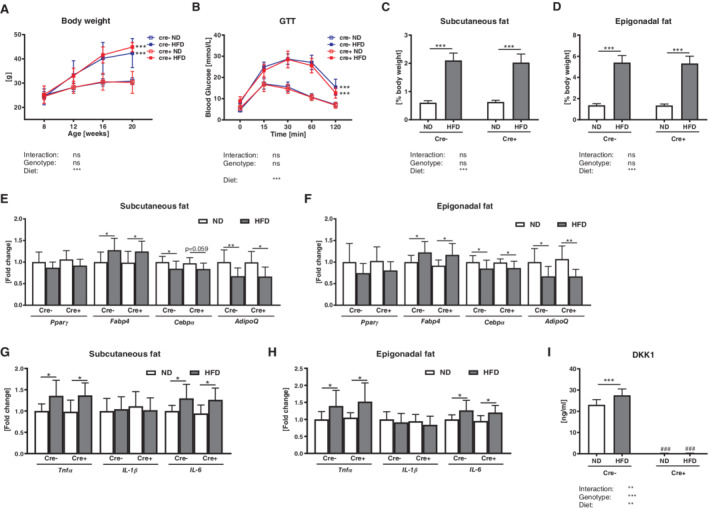
Mice that lack *Dkk1* in osteoprogenitors show similar signs of obesity, despite reduced DKK1 serum levels. Male *Dkk1* ^*fl/fl*^ *;Osx‐Cre* and their Cre‐negative controls were fed a standard (ND) or high‐fat diet (HFD) for 12 weeks. Afterwards (*A*) body weight was assessed and (*B*) a glucose tolerance test (GTT) was carried out. Percentage of body (*C*) subcutaneous and (*D*) epigonadal fat mass was determined. Peroxisome proliferator‐activated receptor gamma (*Pparγ*), fatty acid binding protein (*Fabp4*), CCAAT/enhancer‐binding protein alpha (*Cebpα*), and adiponectin (*AdipoQ*) mRNA expression in (*E*) subcutaneous and (*F*) epigonadal fat mass was analyzed using real‐time PCR analysis. Gene expression of inflammation markers *Tnfα*, *Il‐1β*, and *Il‐6* in (*G*) subcutaneous and (*H*) epigonadal fat mass was analyzed. (*I*) Serum Dickkopf‐1 (DKK1) serum levels were assessed using commercially available ELISAs. Gene expression levels were normalized to *Rpl26*. Data represent the mean ± SD (*n* = 10 to 14/group). Statistical analysis was performed by two‐way ANOVA for the effect of genotype and HFD and the interaction. For weight and GTT area under the curve was determined. *p < 0.05, **p < 0.01, ***p < 0.001 for ND versus HFD. #*p* < 0.05, ##*p* < 0.01, ###*p* < 0.001 versus respective Cre‐negative control.

As the effects of HFD on bone were mitigated in the cortical compartment of global DKK1‐deficient mice, we further elucidated whether osteoprogenitor‐derived DKK1 contributes to a HFD‐induced bone loss. Cre‐negative as well as *Dkk1* ^*fl/fl*^ *;Osx‐Cre* mice gained a similar amount of weight over time when fed a ND or a HFD (Fig. 5 *A*). Also, a HFD decreased glucose tolerance in *Dkk1* ^*fl/fl*^ *;Osx‐Cre* and control mice (Fig. 5 *B*).

A HFD also was associated with an increased percentage of subcutaneous (+68–71%) and epigonadal (+75–79%) fat mass in *Dkk1* ^*fl/fl*^ *;Osx‐Cre* and control mice (Fig. 5 *C*,*D*). The HFD upregulated *Fabp4* expression in both subcutaneous and epigonadal fat tissue, whereas it reduced *AdipoQ* expression (Fig. 5 *E*,*F*). Except for subcutaneous fat tissue of *Dkk1* ^*fl/fl*^ *;Osx‐Cre* mice, *Cebpα* expression was significantly downregulated after a HFD, whereas *Pparγ* expression was not altered. Osteoprogenitor‐specific *Dkk1* deletion did not influence their gene expression when compared with Cre‐negative controls fed a ND. Again, the HFD was associated with an increased expression of *Tnfα* and *Il‐6* in subcutaneous and epigonadal fat tissue, whereas *Il‐1β* expression was not affected (Fig. 5 *G*,*H*).

**Figure 6:**
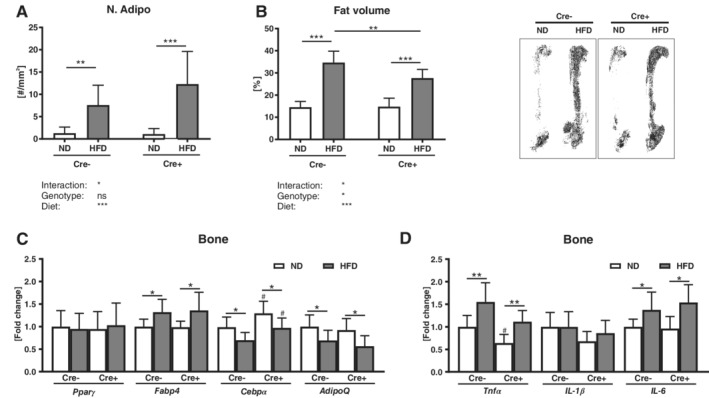
Effects of osteoprogenitor‐specific *Dkk1* deletion on HFD‐induced expansion of bone marrow adipose tissue. *Dkk1* ^*fl/fl*^ *;Rosa26‐CreERT2* and their Cre‐negative controls were fed a normal (ND) or high‐fat diet (HFD) for 12 weeks. The amount of fat in the tibias was assessed by counting (*A*) the number of adipocyte on tartrate‐resistant acid phosphatase‐ (TRAP‐) stained tissue sections and by (*B*) osmium staining of the whole femur. (*C*) Peroxisome proliferator‐activated receptor gamma (*Pparγ*), fatty acid binding protein (*Fabp4*), CCAAT/enhancer‐binding protein alpha (*Cebpα*), and adiponectin (*AdipoQ*) mRNA expression in ulnas was analyzed using real‐time PCR analysis. (*D*) Gene expression of inflammation markers *Tnfα*, *Il‐1β*, and *Il‐6* in ulna was analyzed. Gene expression levels were normalized to *β‐actin*. Data represent the mean ± SD (*n* = 8 to 12/group). Statistical analysis was performed by two‐way ANOVA for the effect of genotype and HFD and the interaction. *p < 0.05, **p < 0.01, ***p < 0.001 for ND versus HFD. #*p* < 0.05, ##*p* < 0.01, ###*p* < 0.001 versus respective Cre‐negative control.

Furthermore, a HFD was associated with increased DKK1 serum levels in Cre‐negative controls, but not in osteoprogenitor‐specific *Dkk1* cKO mice, in which DKK1 serum levels are dramatically reduced (Fig. 6 *I*).^34^ As seen before in global cKO mice, HFD had no impact on SOST serum level (Cre‐negative: ND: 155 ± 13, HFD: 149 ± 17, Cre‐positive: ND: 183 ± 13, HFD: 176 ± 143. However, osteoprogenitor‐specific DKK1 deletion is associated with an increased SOST serum level.

### Osteoprogenitor‐derived DKK1 has a minor influence on bone marrow adiposity

*Dkk1* ^*fl/fl*^ *;Osx‐Cre* and control mice showed both a significant increase in the adipocyte number (+83–91%), adipocyte area (+65–77%), and bone marrow fat mass (+36–48%) after a HFD, albeit increased bone marrow fat was less pronounced in *Dkk1* ^*fl/fl*^ *;Osx‐Cre* mice (Fig. 6 *A*,*B* and Table 2). In both groups, a HFD resulted in an increased expression of *Fabp4*, whereas *Cebpα* and *AdipoQ* expression were reduced in the bone tissue (Fig. 6 *C*). *Pparγ* expression was not altered. *Tnfα* and *Il‐6* expression were increased in both genotypes after a HFD, albeit osteoprogenitor‐specific *Dkk1* cKO mice fed a ND exhibited an overall reduced *Tnfα* expression (Fig. 6 *D*).

**Table 2:** Bone Microstructure and Histological Parameters of Femurs and Tibias of 20‐Week‐Old *Dkk1* ^*fl/fl*^ *;Osx‐Cre* Mice

	Cre‐negative			Cre‐positive					
	ND (*n* = 14)	HFD (*n* = 12)	% change	ND (*n* = 12)	HFD (*n* = 10)	% change	Interaction	Genotype	Diet
*Dkk1* ^*fl/fl*^ *;Osx‐Cre*									
μCT—femur									
BV/TV (%)	5.43 ± 0.96	3.57 ± 1.14	−34%	11.2 ± 2.22***	8.78 ± 1.59***	−21%	0.087	<0.01	<0.001
Tb.N (1/mm)	4.17 ± 0.38	3.45 ± 0.20	−17%	4.92 ± 0.61**	4.31 ± 0.54***	−13%	0.075	<0.01	<0.05
Tb.Th (μm)	39.8 ± 3.33	36.5 ± 2.73	−8%	39.9 ± 3.56	37.4 ± 2.34	−6%	0.755	0.452	<0.001
Tb.Sp (mm)	0.23 ± 0.04	0.26 ± 0.03	+12%	0.19 ± 0.01**	0.20 ± 0.02***	+7%	0.281	0.102	0.093
Histomorphometry—tibia									
Adipo. Ar (mm^2^)	0.70 ± 1.35	2.03 ± 0.60	+65%	0.41 ± 0.71	1.74 ± 1.20	+77%	0.391	0.488	<0.001
BFR/BS (μm^3^/μm^2^/d)	0.26 ± 0.11	0.08 ± 0.10	−57%	0.45 ± 0.06*	0.26 ± 0.19*	−53%	0.920	<0.001	<0.001
MS/BS (%)	25.6 ± 1.89	21.8 ± 1.44	−15%	27.5 ± 1.80	25.2 ± 1.23**	−9%	0.224	<0.001	<0.001
MAR (μm/d)	1.13 ± 0.46	0.57 ± 0.66	−50%	1.63 ± 0.14	1.05 ± 0.77*	−35%	0.974	<0.05	<0.01
N.Oc/B.Pm (#/mm)	7.05 ± 3.16	3.51 ± 3.61	−50%	5.87 ± 3.41	3.12 ± 2.49	−47%	0.383	0.743	<0.001
N.Ob/B.Pm (#/mm)	5.68 ± 1.49	4.44 ± 1.03	−33%	5.71 ± 1.34	4.72 ± 1.28	−17%	0.659	0.631	0.177

Data represent the mean ± SD (*n* = 10 to 14/group). Statistical analysis was performed using two‐way ANOVA. *p* Values from ND versus HFD. *p < 0.05, **p < 0.01, ***p < 0.001 versus respective Cre‐negative control. BV/TV = bone volume/total volume; Tb.N = trabecular number; Tb.Th = trabecular thickness; Tb.Sp = trabecular separation; Adipo. Ar = adipocyte area; BFR/BS = bone formation rate/bone surface; MS/BS = mineralizing surface/bone surface; MAR = mineral apposition rate; N.Oc/B.Pm = number of osteoclasts/bone perimeter; N.Ob/B.Pm = number of osteoblasts/bone perimeter.

### Osteoprogenitor‐specific DKK1 deletion protects against obesity‐induced cortical bone loss, but not trabecular bone loss

**Figure 7:**
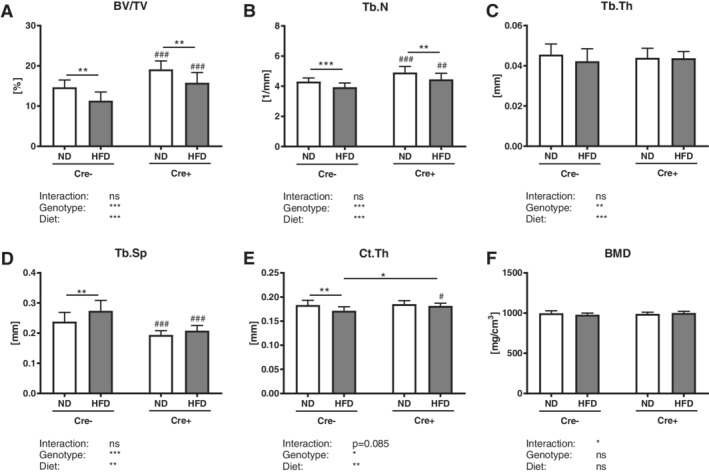
Osteoprogenitor‐specific *Dkk1* deletion does not protect against obesity‐induced bone loss. The fourth vertebral body of 20‐week‐old male *Dkk1* ^*fl/fl*^ *;Osx‐Cre* (Cre‐positive and Cre‐negative) mice after 12 weeks of normal (ND) or high‐fat diet (HFD) was analyzed by μCT. (*A*) Trabecular bone volume per total volume (BV/TV) and (*B*) trabecular number (Tb.N), (*C*) trabecular thickness (Tb.Th), and (*D*) trabecular separation (Tb.Sp) of the fourth vertebral body. Cortical compartment of the femur was analyzed by μCT to examine (*E*) cortical thickness (Ct.Th) and (*F*) BMD of the femur. Data represent the mean ± SD (*n* = 10 to 14/group). Statistical analysis was performed by two‐way ANOVA for the effect of genotype and a HFD and the interaction. *p < 0.05, **p < 0.01, ***p < 0.001 for ND versus HFD. #*p* < 0.05, ##*p* < 0.01, ###*p* < 0.001 versus respective Cre‐negative control.

Although *Dkk1* ^*fl/fl*^ *;Rosa26‐CreERT2* mice were protected against obesity‐induced bone loss, *Dkk1* ^*fl/fl*^ *;Osx‐Cre* mice and their Cre‐negative controls lost a similar amount of vertebral trabecular bone after a HFD (−17–23%; Fig. 7 *A*). Furthermore, both showed a significant reduction of trabecular number (−9%), no changes in trabecular thickness, whereas trabecular separation was significantly increased (+7–13%) after a HFD (Fig. 7 *B*–*D*). Similar results were found in the femurs of these mice (Table 2). However, only Cre‐negative HFD mice exhibited a significantly reduced cortical thickness (−6%), whereas cortical bone was not affected in *Dkk1* ^*fl/fl*^ *;Osx‐Cre* mice (Fig. 7 *E*). Both groups showed no changes in cortical BMD (Fig. 7 *F*).

**Figure 8:**
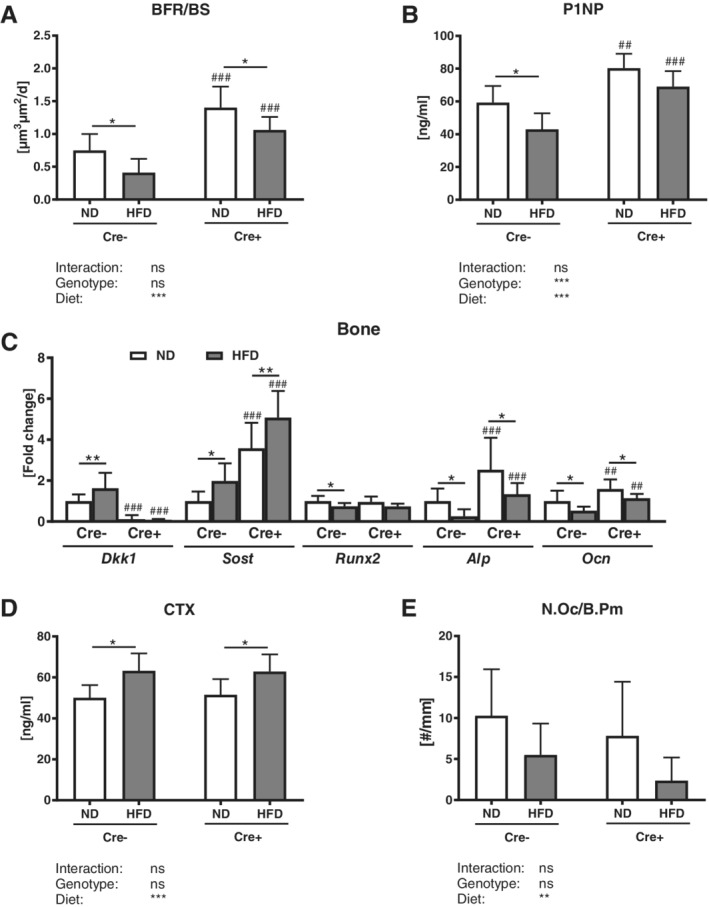
Osteoprogenitor‐specific *Dkk1* deletion does not protect against obesity‐induced changes in bone formation and bone resorption. Histomorphometric and serum osteoblast and osteoclast parameters of 20‐week‐old male *Dkk1* ^*fl/fl*^ *;Osx‐Cre* (Cre‐positive and Cre‐negative) mice after 12 weeks of normal (ND) or high‐fat diet (HFD) were analyzed. (*A*) Quantification of serum procollagen type 1 amino‐terminal propeptide (P1NP) was performed by ELISA. Histomorphometric analysis of calcein double staining of tibias was performed to determine (*B*) the bone formation rate/bone surface (BFR/BS). (*C*) Dickkopf‐1 (*Dkk1*), sclerostin (*SOST*), runt‐related transcription factor 2 (*RUNX2*), alkaline phosphatase (*Alp*), and osteocalcin (*Ocn*) mRNA expression in ulna bone tissue was analyzed using real‐time PCR analysis. Gene expression levels were normalized to *β‐actin*. (*D*) Serum CTx was measured using ELISA. (*E*) Tartrate‐resistant acid phosphatase (TRAP) staining was used to determine the number of osteoclasts/bone parameter (N.Oc/b.pm) in tibias. Data represent the mean ± SD (*n* = 10 to 14/group). Statistical analysis was performed by two‐way ANOVA for the effect of genotype and HFD and the interaction. *p < 0.05, **p < 0.01, ***p < 0.001 for ND versus HFD. #*p* < 0.05, ##*p* < 0.01, ###*p* < 0.001 versus respective Cre‐negative control.

Even though only Cre‐negative mice exhibited significantly reduced P1NP serum levels after a HFD, both groups showed a reduced bone formation rate (−24–54%) and mineral surface per bone surface (−21%), whereas the MAR was not altered (Fig. 8 *A*,*B* and Supplemental Fig. S2). The HFD increased local *Dkk1* expression only in the bones of Cre‐negative mice (Fig. 8 *C*). *Sost* expression was significantly increased in both HFD groups, with *Dkk1* ^*fl/fl*^ *;Osx‐Cre* mice showing an overall higher *Sost* expression compared with their Cre‐negative controls (Fig. 8 *C*). *RUNX2* expression was only significantly decreased in control mice fed HFD, whereas *Alp* and *Ocn* were reduced in both groups. The *Dkk1* ^*fl/fl*^ *;Osx‐Cre* mice showed a higher *Alp* and *Ocn* expression compared with littermate controls. A HFD increased CTx serum levels in *Dkk1* ^*fl/fl*^ *;Osx‐Cre* and Cre‐negative control mice, whereas the number of osteoclasts was not altered (Fig. 8 *C*,*D*).

## Discussion

Obesity is becoming a global epidemic, which is associated with chronic low‐grade inflammation and metabolic dysregulation including alterations in bone metabolism and strength.^3^, ^4^ The underlying mechanisms, however, remain poorly understood. Recently, we detected an increased amount of DKK1‐expressing osteoblasts and elevated DKK1 serum levels in obese mice.^20^ Thus, in this study we tested the hypothesis that DKK1 plays a role in obesity‐induced negative effects on bone.

First, our data support the notion that HFD induces bone loss in mice, which is controversially discussed in the literature. Although most studies also report bone loss in young, adolescent, and mature obese rodents on a HFD,^18^, ^42^, ^43^, ^44^ some find no alterations^17^, ^45^, ^46^ or even increased bone mass.^47^ These discrepancies may be provoked by different age and sex, varying intervention times (6 to 12 weeks), and diet compositions (45% to 60% fat in chow), highlighting the importance of detailed investigations and standardized procedures.

Our key finding was that global and osteogenic deletion of *Dkk1* protected mice from obesity‐induced cortical, but not trabecular bone loss. Distinct effects of some components of the Wnt signaling pathway on cortical versus trabecular bone have been described previously. For example, Wnt16 has distinct effects only on cortical, but not trabecular bone, suggesting a differential homeostatic regulation between the cortical and trabecular bone compartments.^48^, ^49^ Furthermore, it should be mentioned that although a HFD has detrimental effects on cancellous bone mass, the effects on cortical bone mass in rodents are quite diverse. Although some studies have reported increases^47^, ^50^ or decreases in cortical bone mass,^37^, ^51^ the majority of studies report no difference between normal and HFD.^52^, ^53^, ^54^ In our study, cortical bone loss was not profound, but consistent among the experiments. Thus, though under homeostatic conditions *Dkk1* is critical for both the cortical and trabecular compartments,^34^ in obesity it drives cortical bone loss.

As suppression of bone formation is one of the main mechanisms of HFD‐induced bone loss, we speculated that osteogenic deletion of DKK1 would ameliorate bone loss. However, a HFD still reduced trabecular bone formation parameters in *Dkk1* ^*fl/fl*^ *;Osx‐Cre* mice, which, as previously published, were generally higher in *Dkk1* ^*fl/fl*^ *;Osx‐Cre* mice compared with Cre‐negative controls.^34^ This indicates that blocking osteogenic DKK1 is not sufficient to protect from HFD‐induced bone loss and raises the question, which cell type may then be involved. DKK1 is produced by numerous cells, including osteogenic cells, platelets, adipocytes, fibroblasts, and vascular cells.^27^, ^55^, ^56^, ^57^ As obesity is associated with an expansion of adipose tissue and adipocytes express DKK1, it may be envisaged that DKK1 produced from this cell type may play a major role. In fact, a previous study by Gao and colleagues found an increased expression of *Dkk1* in epididymal adipose tissue of HFD‐fed mice.^30^ Furthermore, the natural compound embelin efficiently inhibited adipogenesis and lipogenesis in vitro, which was likely caused by an activation of canonical Wnt signaling, as embelin treatment attenuated the induction of *Dkk1* in adipose tissue in HFD‐fed mice.

Previously, we detected increased DKK1 serum levels in obese mice, whereas the serum concentration of another important Wnt inhibitor SOST was unaffected.^20^ Other studies, however, have shown increased skeletal SOST levels under HFD conditions, which we also confirm in this study. Moreover, because of the well‐documented negative feedback loop between DKK1 and SOST,^58^, ^59^ skeletal expression of SOST was also higher in Cre‐positive mice. Nonetheless, the increase in skeletal SOST levels was not sufficient to balance the lack of *Dkk1* expression and therefore did not alter bone mass. Interestingly, serum SOST levels were found to be positively correlated with abdominal and gonadal fat, as well as with biochemical markers related to metabolic disease in postmenopausal women.^60^ Additionally, SOST knockout or antibody treatment results in reduced adipose tissue accumulation, which is associated with increased insulin sensitivity and improved metabolic parameters.^61^ This indicates that Wnt inhibitors are influenced by obesity and may have endocrine functions between adipose tissue and the skeleton. A human study showed that DKK1 serum levels are not correlated with insulin levels or insulin resistance, but to BMI, waist circumference, and total‐body adiposity.^62^ Therefore, DKK1 is discussed as a potential novel marker for adiposity.^62^ However, we did not previously find any differences between serum DKK1 levels in lean, overweight, and obese individuals.^20^ It should be mentioned that the investigated human cohort did not include patients diagnosed with type 2 diabetes, whereas the HFD model used in this study is known to result in prediabetes, including an impaired glucose tolerance as well as insulin resistance.^63^ We therefore assume that DKK1 could be influenced by insulin sensitivity as obese mice with impaired glucose tolerance and insulin sensitivity showed increased DKK1 serum levels in the current and previous study.^20^ Deletion of β‐catenin in pancreatic progenitors led to a decreased β‐cell mass and impaired glucose tolerance.^64^ Furthermore, this loss of β‐catenin made the mice resistant to HFD‐induced obesity and insulin resistance, implicating an important role of β‐catenin in the regulation of metabolism and energy homeostasis. Thus, Wnt signaling may modulate the susceptibility to diabetes by acting on different tissues.

Feeding mice a HFD resulted in the accumulation of subcutaneous and epigonadal fat depots as well as bone marrow adiposity in Cre‐negative mice, which validates the existing literature.^17^, ^20^, ^65^ The amount of body fat also increased to a similar extent in Cre‐positive *Dkk1* ^*fl/fl*^ *;Osx–Cre* mice, along with similar dysregulations in adipogenic markers, which are typically associated with the development of insulin resistance.^66^, ^67^, ^68^ As obesity leads to an increased secretion of proinflammatory cytokines such as TNFα and IL‐6, we also investigated them in this study.^69^, ^70^ These cytokines are known to promote the development of insulin resistance, type 2 diabetes mellitus, and elevate osteoclastogenesis.^69^, ^71^, ^72^, ^73^ The expression of *Tnfα* in bone was less pronounced in bone of ND‐fed Cre‐positive mice of both genotypes compared with Cre‐negative controls, whereas a HFD increased its expression level to the same amount than in the Cre‐negative HFD group.

Our study has potential limitations. First, a tamoxifen‐inducible Cre line was used to delete *Dkk1* postnatally, even though tamoxifen has been shown to have direct effects on bone homeostasis.^74^, ^75^ However, as global deletion of *Dkk1* is lethal, an inducible transgenic mouse model represents the currently best available option. Moreover, both Cre‐negative and Cre‐positive mice received tamoxifen, thus exposing both experimental groups to the same bias. Also, the Osx–Cre has known limitations including targeting other cell types than just osteoprogenitors and an intrinsic bone phenotype when not suppressed during embryogenesis.^76^ However, we suppressed Cre activity during embryogenesis and during early life using doxycycline and found no difference in the bone phenotype using this protocol.^34^ Also, according to our previous study,^34^ we expected stronger effects of *Dkk1* on trabecular bone, and thus sectioned the bones in such a way that it is not possible to obtain data on bone formation in cortical bone. Such data might have provided further insights into how DKK1 protects from obesity‐induced cortical bone loss. Finally, a HFD induces bone loss in mice, which is not observed in humans.^20^ Thus, this model does not fully recapitulate impaired bone strength in obese individuals. Nonetheless, the impact of high glucose and insulin resistance on bone can be studied well in this model, but requires further validation in humans.

In summary, we showed that *Dkk1* from osteogenic cells has no effect on metabolic parameters, but may contribute to the expansion of bone marrow fat in obesity. More importantly, osteogenic *Dkk1* appears to drive cortical, but not trabecular bone loss caused by obesity.

## Disclosures

All authors state that they have no conflicts of interest.

## Supporting information


**Appendix S1.** Supporting information.
