# Supplementary material for: Contributions of Dickkopf‐1 to Obesity‐Induced Bone Loss and Marrow Adiposity
Source: JBMR Plus. 2020 Apr 28;4(6):e10364. doi: 10.1002/jbm4.10364 (PMC7285751; doi:10.1002/jbm4.10364)
Supplement: Supplementary file 1 — Appendix S1. Supporting information. [file JBM4-4-e10364-s001.doc]

**Supplemental Material**

**Materials and Methods**

*Intraperitoneal glucose tolerance test*

Groups of male ND or HFD fed mice were used for intraperitoneal glucose injection. After 16 hours fasting, blood glucose levels were measured and D-glucose (2 g/kg body weight) was administered. Blood samples were taken from the tail vein at indicated times. Blood glucose levels were detected at 15, 30, 60, 90, 120 min and blood glucose concentration was determined using a glucometer (ACCU CHEK Aviva III; Roche Diabetes Care, Mannheim, Germany).

### *Blood and serum analysis*

Blood glucose concentration was measured during the experiment in tail blood (ACCU CHEK Aviva III; Roche Diabetes Care, Mannheim, Germany). After sacrifice, blood was collected by punctuation of the heart, centrifuged and plasma was frozen at −80 °C. Serum levels of DKK1 (R&D, Minneapolis, Minnesota USA), C-terminal telopeptide (CTX), and type 1 procollagen amino-terminal-propeptide (P1NP) (CTX and P1NP: Immundiagnostik Systems, Bensheim, Germany) were detected using an immunoassay kit according to the manufacturer's protocol.

*Assessment of bone mass, microarchitecture and fat content*

Bones were extracted and fixed in 4% paraformaldehyde (PFA, Carl Roth, Karlsruhe, Germany) and dehydrated in 80% ethanol. By using the μCT vivaCT40 (Scanco Medical AG, Brüttisellen, Switzerland), the femur and third vertebral body were analyzed as previously described (1). The μCT parameters were reported according to international guidelines (2).

For assessment of the [bone marrow](https://www.sciencedirect.com/topics/medicine-and-dentistry/bone-marrow) fat content, the intact fixed femora were decalcified (Osteosoft ®, Merck) for one week. After scanning the femora with the μCT to ensure complete [decalcification](https://www.sciencedirect.com/topics/medicine-and-dentistry/decalcification), the femora were briefly washed in PBS and stained for 1 h with 2% [osmium tetroxide](https://www.sciencedirect.com/topics/medicine-and-dentistry/osmium-tetroxide) (Electron Microscopy Science, Hatfield, Pennsylvania, USA) to visualize fat droplets in the [adipocytes](https://www.sciencedirect.com/topics/medicine-and-dentistry/adipocyte) (1). Femora were then transferred into PBS and scanned (10.5 µm voxel size, 300 ms integration time, 70 kVP) and analyzed for their fat volume using the vivaCT40.

*Bone histology and histomorphometry*

Five and two days before sacrifice, all mice received an intraperitoneal injection of [calcein](https://www.sciencedirect.com/topics/medicine-and-dentistry/calcein) (20 mg/kg, Merck, Darmstadt, Germany). Both tibiae were fixed in PFA and dehydrated with ethanol as reported previously (1). Left [tibia](https://www.sciencedirect.com/topics/medicine-and-dentistry/tibia) were embedded in [methacrylate](https://www.sciencedirect.com/topics/medicine-and-dentistry/methacrylic-acid-methyl-ester) (Technovit 9100, Heraeus Kulzer, Germany) and cut into sections to analyze the fluorescent calcein labels on the trabecular bone. [Bone formation](https://www.sciencedirect.com/topics/medicine-and-dentistry/ossification) rate per bone surface (BFR/BS), mineralized surface per bone surface (MS/BS), and mineral apposition rate (MAR) were assessed using Osteomeasure software (OsteoMetrics, Atlanta, Georgia, USA) (3). The osteoclast number per bone perimeter (N.Oc/B.Pm), osteoblast number per bone perimeter (N.Ob/B.Pm), were analyzed by TRAP staining on decalcified sections (1). Bone sections were evaluated for adipocyte area (Adipo. Ar: area equals the size of the adipocyte ghosts) and adipocyte number (N.Adipo).

### *RNA isolation, RT, and quantitative real time PCR (qRT-PCR)*

Total RNA from bone samples (ulnae) was isolated using Trifast (PEQLAB, Radnor, Pennsylvania USA) following the manufacturer’s instructions. RNA of visceral subcutaneous and epigonadal adipose fat was isolated using the peqGOLD Total RNA Kit following the manufacturer’s instructions. Briefly, using SiLibeads, 2.6-3.3 mm (Sigmund Lindner, Warmensteinbach, Germany), 50 mg of fat tissue was first lysed and homogenized with the homogenizer Precellys 24 (Bertin Technologies SAS, Montigny-le-Bretonneux, France) two times for 30 s at 5000 rpm with 10 seconds pause. Afterwards, 500 ng RNA were reverse transcribed using Superscript II (Invitrogen, Carlsbad, California, USA) und subsequently used for SYBR green-based real-time PCR analysis using a standard protocol (Life Technologies, Carlsbad, California, USA). Used primers are listed in **Supplementary Table 1.** Quantitative gene expression was calculated from the standard curve of cloned cDNA and was normalized to the unregulated reference genes β-Actin (bones) or *Rpl26* (fat tissue) as indicated.

### *Statistical analysis*

Using GraphPad Prism 7.0 software, we tested normal distribution by Shapiro-Wilk normality test and afterwards analyzed the effects of [Cre-](https://www.sciencedirect.com/topics/medicine-and-dentistry/lipid-diet) and Cre+ by two-way ANOVA with Tukey post-hoc test. For weight and glucose tolerance test area under the curve was determined. Results are given as the mean ± standard deviation (SD) and P values of <0.05 were considered statistically significant.

**References**

1. Picke AK, et al. (2016) Bone defect regeneration and cortical bone parameters of type 2 diabetic rats are improved by insulin therapy. *Bone*. doi:10.1016/j.bone.2015.06.001.

2. Bouxsein ML, et al. (2010) Guidelines for assessment of bone microstructure in rodents using micro-computed tomography. *J Bone Miner Res* 25(7):1468–1486.

3. Dempster DW, et al. (2013) Standardized nomenclature, symbols, and units for bone histomorphometry: a 2012 update of the report of the ASBMR Histomorphometry Nomenclature Committee. *J Bone Miner Res* 28(1):2–17.

**Supplemental Tables**

**Supplementary Table 1.** Murine primer sequences for RT-PCR.

| **Target gene** | **Primer sequences 5’ – 3’** | |
| --- | --- | --- |
|  | **Sense** | **Antisense** |
| AdipoQ | TGGAGAGAAGGGAGAGAAAGG | TGAGCGATACACATAAGCGG |
| Alp | CTACTTGTGTGGCGTGAAGG | CTGGTGGCATCTCGTTATCC |
| βAct | GATCTGGCACCACACCTTCT | GGGGTGTTGAAGGTCTCAAA |
| Cebpα | CTGAGAGCTCCTTGGTCAAG | GAATCTCCTAGTCCTGGCTTG |
| Dkk1 | GCC TCC GAT CAT CAG ACG GT | GCA GGT GTG GAG CCT AGA AG |
| Fabp4 | ATGTGTGATGCCTTTGTGGGAAC | TCATGTTGGGCTTGGCCATG |
| IL-1β | ACAAGGAGAACCAAGCAACG | GCCGTCTTTCATTACACAGG |
| IL-6 | ACTTCCATCCAGTTGCCTTC | ATTTCCACGATTTCCCAGAG |
| Ocn | GAAGACCGCCTACAAACG | TTTGGAGCTGCTGTGACATC |
| Pparɣ | CATGGTTGACACAGAGTGC | CTTGCATCCTTCACAAGCATG |
| Rpl26 | GGACCCGAGAAGACCTCCTT | GCACATCACTCAGAATTTCAATGG |
| Runx2 | CTCCAAGACCCTAAGAAACCG | TCTCTCAGATACCATGGGTGC |
| Sost | CGTGCCTCATCTGCCTACTT | TGACCTCTGTGGCATCATTC |
| Tnfα | GCTGAGCTCAAACCCTGGTA | CGGACTCCGCAAAGTCTAAG |

AdipoQ = Adiponectin, Alp = alkaline phosphatase, βAct = beta actin, Cebpα = CCAAT/enhancer-binding protein alpha, Dkk1 = dickkopf-1, Fabp4 = fatty acid binding protein 4, Ocn = osteocalcin, Pparɣ = peroxisome proliferator-activated receptor gamma, Rpl26 = ribosomal protein L26, Runx2 = runt-related transcription factor 2, Sost = sclerostin

**Supplementary Figure 2.** Histological parameters of the vertebrae of 20-week-old *Dkk1^fl/fl^; Rosa26-CreERT2 and Dkk1^fl/fl^;Osx-Cre mice*.

|  | **Cre-** | | | **Cre+** | | | |  |  |  |
| --- | --- | --- | --- | --- | --- | --- | --- | --- | --- | --- |
| **Vertebrae** | **ND**  **(n=12-14)** | **HFD**  **(n=10-12)** | **% change** | **ND**  **(n=8-12)** | **HFD**  **(n=10)** | **% change** | | **Interaction** | **Genotype** | **Diet** |
| ***Dkk1^fl/fl^; Rosa26-CreERT2*** | | |  |  |  | | |  |  |  |
| *Histomorphometry* | |  |  |  |  |  | |  |  |  |
| MS/BS [%] | 30.6 ± 3.45 | 25.3 ± 2.93 | -17% | 32.5 ± 4.16 | 29.7 ± 2.62 | | -9% | 0.291 | <0.05 | <0.01 |
| MAR [µm/d] | 0.98 ± 0.24 | 0.83 ± 0.43 | -15% | 1.85 ± 0.37^###^ | 1.64 ± 0.44^###^ | | -11% | 0.807 | <0.001 | 0.171 |
| N.Ob/B.Pm [#/mm] | 4.50 ± 1.62 | 1.38 ± 0.56 | -70% | 6.04 ± 2.48 | 3.26 ± 1.58 | | -46% | 0.763 | <0.05 | <0.001 |
| ***Dkk1^fl/fl^;Osx-Cre*** | |  |  |  |  | |  |  |  |  |
| *Histomorphometry* | |  |  |  |  | |  |  |  |  |
| MS/BS [%] | 26.1 ± 3.15 | 21.1 ± 2.58 | -19% | 27.6 ± 4.80 | 21.8 ± 2.84 | | -21% | 0.754 | 0.375 | <0.001 |
| MAR [µm/d] | 1.43 ± 0.19 | 1.25 ± 0.24 | -12% | 2.06 ± 0.28^###^ | 1.88 ± 0.34^###^ | | -9% | 0.993 | <0.001 | 0.067 |
| N.Ob/B.P [#/mm] | 5.52 ± 2.17 | 3.45 ± 1.06 | -37% | 7.25 ± 2.57 | 4.62 ± 1.34 | | -36% | 0.051 | <0.05 | <0.01 |

MS/BS = mineralizing surface/bone surface, MAR = mineral apposition rate, and N.Ob/B.Pm = number of osteoblasts/bone perimeter. Data represent the mean ± SD. Statistical analysis was performed using two-way ANOVA. P-values from ND vs HFD. #p<0.05, ##p<0.01, ###p<0.001 vs respective Cre-negative control.
